# Beetroot Juice Produces Changes in Heart Rate Variability and Reduces Internal Load during Resistance Training in Men: A Randomized Double-Blind Crossover

**DOI:** 10.3390/nu14235119

**Published:** 2022-12-02

**Authors:** Jose Manuel Jurado-Castro, David Casanova-Rodriguez, Julian Campos-Perez, Francisco Jesus Llorente-Cantarero, Candelaria Alonso De La Florida-Villagran, Víctor Manuel Diaz-Bernier, Antonio Ranchal-Sanchez

**Affiliations:** 1 Metabolism and Investigation Unit, Maimonides Biomedical Research Institute of Cordoba (IMIBIC), Reina Sofia University Hospital, University of Cordoba, 14004 Cordoba, Spain; 2 CIBER Fisiopatología de la Obesidad y Nutrición (CIBEROBN), Instituto de Salud Carlos III, 28029 Madrid, Spain; 3 Ciencias De La Actividad Física y El Deporte, Escuela Universitaria de Osuna (Centro Adscrito a la Universidad de Sevilla), 41640 Osuna, Spain; 4 Department of Nursing, Pharmacology and Physiotherapy, Faculty of Medicine and Nursing, University of Cordoba, 14071 Cordoba, Spain; 5 Department of Food Science and Technology, Campus Universitario de Rabanales, University of Cordoba, 14004 Cordoba, Spain; 6 Department of Specific Didactics, Faculty of Education, University of Cordoba, 14004 Cordoba, Spain; 7 Grupo De Investigación Clínico Epidemiológica De Atención Primaria, Maimonides Biomedical Research Institute of Cordoba (IMIBIC), Reina Sofia University Hospital, University of Cordoba, 14004 Cordoba, Spain

**Keywords:** heartbeat, heart rate control, dietary supplement, nitric oxide, autonomic nervous system, beta vulgaris

## Abstract

Beetroot juice (BJ) has been used as a sport supplement, improving performance in resistance training (RT). However, its effect on the modulation of the autonomic nervous system has not yet been widely studied. Therefore, the objective of this randomized double-blind crossover study was to assess the effect of acute BJ supplementation compared to placebo in blood pressure (BP), heart rate (HR), heart rate variability (HRV) and internal load during RT measure as Root Mean Square of the Successive Differences between adjacent RR intervals Slope (RMSSD and RMSSD-Slope, respectively). Eleven men performed an incremental RT test (three sets at 60%, 70% and 80% of their repetition maximum) composed by back squat and bench press with. HR, HRV and RMSSD-Slope were measured during and post exercise. As the main results, RMSSD during exercise decrease in the BJ group compared to placebo (*p* = 0.023; ES = 0.999), there were no differences in RMSSD post-exercise, and there were differences in RMSSD-Slope between groups in favor of the BJ group (*p* = 0.025; ES = 1.104) with a lower internal load. In conclusion, BJ supplementation seems to be a valuable tool for the reduction in the internal load of exercise during RT measured as RMSSD-Slope while enhancing performance.

## 1. Introduction

Beetroot juice (BJ) and its influence on sport performance has been studied [1], showing a great interest in the sport nutrition field and the sport performance in different disciplines, and not least, being also interesting on general population health reducing different cardiovascular and coronary diseases [1,2]. Mainly, the effects of BJ and its interest on the field of health, sport nutrition and sport performance are mostly based by the principal active principle present of BJ supplementation, which is the dietary inorganic nitrate (NO_3_^−^) [3]. After its ingestion, the NO_3_^−^ goes through a complex digestion and convoluted metabolic pathways. It is first absorbed by the salivary circulation and subsequently reduced to nitrite (NO_2_) by the action of nitrate reductase facultative anaerobic bacteria present at the dorsal surface of the tongue [4]. Then, at the stomach, NO_2_ is decomposed into nitric oxide (NO) and finally reaches the plasma and systemic circulation [5,6].

In sport performance, the physiological effects of BJ supplementation and NO could be summed up in a dilation of the vascular endothelium [7,8], causing a vasodilator effect and reducing the blood pressure (BP) [9,10]. In addition, these physiological effects are interesting for sport performance because it could increase muscle blood flow [11], alter and improve lactate removal in the exercise [12]. In addition, the intake of NO_3_^−^ effects has been proved in different endurance sports, where cardiovascular system has an important role in performance [1,7,13], showing a lower oxygen consumption (VO2) during exercise [1] with an improvement in adenosine triphosphate (ATP) synthesis [14].

Nevertheless, in strength sports or resistance training (RT), BJ supplements have received less attention [3]. However, research has shown BJ as an effective ergogenic aid in RT, observing an increase in muscle strength, explosive force and muscular endurance due to better ATP utilization, increased blood volume, and therefore, oxygen during exercise [15,16,17,18,19].

In the cardiovascular system, NO regulates various functions such as contractile force, myocardial relaxation, mitochondrial respiration and coronary perfusion [20], and it clearly has a cardioprotective role in pathologies such as myocardial infarction and heart failure [21]. In animal models of cardiac ischemia–reperfusion, treatment with NO_2_ produced a clear cardioprotective effect [22,23,24,25]. However, the results of human studies are not so obvious [26].

Normally, a healthy heart is not a regular metronome with the same interval between heartbeats, but it experiences variations of the normal rhythm of the heart. To measure and control these different oscillations of the period between consecutive heartbeats, heart rate variability (HRV) is a proved valuable tool [27]. HRV can be used as a mirror of the cardiorespiratory control system and parasympathetic function of the autonomic nervous system (ANS), being a useful signal for understanding the status of the ANS [28]. According to a meta-analysis [29], HRV data obtained in men and women cannot be treated equally and that studies need to characterize these differences. In autonomic control of the heart indexed by HRV measures, it has been observed that women showed a significantly lower mean RR interval, showing greater vagal activity [30].

HRV has a direct relationship between sport performance and physical activity and their physiological effects, decreasing with stress activities such as exercise and when respiratory increases [31]. Recently, HRV has been considered useful to determine the internal load of physical activity [32], evaluating the modulation of the sympathetic and parasympathetic system, specifically, to know the activation of the parasympathetic system in the athlete’s recovery [33,34,35,36]. Using HRV, certain data obtained in his measurement, such as the Root Mean Square of the Successive Differences between adjacent RR intervals (RMSSD), could be one of the best reliable measures of parasympathetic activity. A measurement in a short period of time is sufficient [37]. In this way, Naranjo Orellana et al. have determined the measure of internal load based on the recovery of the RMSSD-Slope on 30 min post-exercise to monitor the effect of workloads and fatigue caused by exercise [32].

Although an electrocardiogram, measured by measurement instruments such as the Holter system, would be the reference method for assessing HRV [38], it not suitable for daily measurement and sport. In this regard, new technologies such as wearable devices [39,40,41,42] and smartphone applications [43,44] have taken the spotlight, becoming the reference method for measuring RR intervals. Chest straps such as the Polar H10 (Polar Inc., Kempele, Finland) can be considered the gold standard for RR interval assessments if intense activities with strong body movements are investigated. Polar H10 has demonstrated the validity of for the detection of RR intervals in a wide range of physical activities and sports [39]. HRV parameters and several nonlinear parameters can be further interpreted and analyzed using advanced HRV analysis software such as Kubios HRV (University of Eastern Finland, Kuopio, Finland) [45]. This software supports several input data formats for electrocardiogram data and beat-to-beat RR interval data.

Despite the knowledge of the ergogenic component of BJ and the changes produced in BP after its consumption pre-exercise, certain physiological post-exercise recovery effects have not been evaluated during RT, especially in relation to HR and HRV, which is a critical period in which various modifications occur, including changes in autonomic modulation [46], which can promote an environment conducive to the development of abnormal alteration in both BP, HR and HRV [46,47]. Thus, due the possible effects of BJ supplementation in addition to RT in cardiovascular system, the purpose of this study was to investigate the possible effects of BJ acute supplementation during RT in BP and the variations in HR, HRV and internal load during RT measured by changes in HRV in man. We hypothesized that the BJ consumption could produce changes in the HRV and produce a reduction in the internal load during RT.

## 2. Materials and Methods

### 2.1. Design

The study was conducted according to the CONSORT statement (Supplemental Material S1) with a double-blind, randomized crossover trial design. The experimental procedure was carried out in 3 visits, with a difference of one week between visits. Body composition was assessed at the first visit in addition to a familiarization protocol and 1RM tests. In the second and third visits, the participants carried out the experimental supplementation protocol (BJ consumption or placebo). In order to standardize the influence of the circadian rhythm, and the possible variation of muscle strength and power [48,49], the visits took place at the same time of day in the morning (±0.5 h) with a temperature of 24 °C ± 1 °C.

### 2.2. Participants

The inclusion/exclusion criteria to recruit trained men were: (a) 18–30 years old; (b) experience of more than 2 years in RT; (c) familiarization with the back squat and bench press exercises; (d) abstinence from the consumption of nutritional supplements or anabolic substances for three months before the study; and (e) absence of musculoskeletal injuries. These criteria were verified through personal interviews.

All participants were informed through an information sheet and a signed written consent before the start of the investigation in accordance with the Declaration of Helsinki [50]. The protocol was approved by Portal de Ética de la Investigación Biomédica de Andalucía ethics committee (protocol code: BEETROOT JUICE; reference 4284).

### 2.3. Anthropometry and Body Composition

During the first visit, anthropometric measurements were made according to the protocol of the International Society for the Advancement of Kinanthropometry (ISAK). Body composition was reported using bioelectrical impedance (MC-780MA; Tanita) and height was recorded with a stadiometer (portable stadiometer; Seca 214) [51].

### 2.4. Study Interventions

#### 2.4.1. Familiarization Protocol, One-Repetition Maximum Testing and Resistance Training Performance (Back Squat and Bench Press)

Before the test, participants performed a familiarization by performing a maximum explosive speed in the concentric movement for the back squat and bench press exercises. The participants lifted 20 kg on a Smith machine (Technogym, Barcelona, Spain) for a total of three repetitions controlling the technique movement of back squat. They received correction by research if necessary.

During the familiarization and 1RM test, the execution velocity was controlled using a linear position transducer (v.4.1, Speed4Lift; Madrid, Spain) used in previous studies [52,53]. A previous testing protocol to find the 1RM load in back squat and bench press was realized [54].

Back squat and bench press were performed on the same day, and the protocol was replicated for both. Test specifications were published previously by Ranchal-Sanchez et al. (2020) [52].

Results regarding performance in back squat and bench press as well as RPE have been published by Ranchal-Sanchez et al. (2020), finding an overall better performance in the RT with the BJ consumption, improving the muscular resistance without differences in ratings of perceived exertion (RPE) [52].

#### 2.4.2. Supplementation Protocol

First, 70 ml of BJ (BEET It Sport^®^; James White Drinks Ltd., Ipswich, UK) with 6.4 mmol·L^−1^ or 400 mg NO_3_^−^ per serving [55] or 70 mL of blackcurrant drink without NO_3_^−^ as a placebo (Capri-Sun, Uxbridge, UK) were taken 120 min before of each visit [1].

Participants completed a 24 h dietary recall, on the day prior to the first visit, as a tool for athletes to replicate their diet [17]. Moreover, they also received nutritional guidelines based on the exchange of food groups to guarantee that 48 h before each visit, they followed a similar diet composed of 60% carbohydrates, 30% lipids and 10% proteins [14,56,57,58], and a list of foods rich in NO_3_^−^ (e.g., beetroot, celery, or spinach) or rich in caffeine that they could not consume.

Twenty-four hours before the experimental visits, the participants were instructed to refrain from brushing their teeth and the use of mouthwash, and the participants had to sleep at least 8 h to ensure optimal hydration and rest during the study period.

### 2.5. Study Outcomes

#### 2.5.1. Blood Pressure

For testing blood pressure, diastolic (DBP) and systolic (SBP) blood pressure in the non-dominant arm were measured (OMROM, HEM-7200-E2 device) at the beginning and the RT post-exercise, measuring three times to dismiss possible measurement failures [59].

#### 2.5.2. Heart Rate and Heart Rate Variability Measurement

During training and recovery, HR was monitored with a Polar H10 heart rate monitor. As the data receiver, the Polar Beat application was used in dual Bluetooth connection to record HR (HR average and maximum HR of the session), allowing a second connection to be made to measure HRV, on another mobile device with the Elite HRV application, which was previously validated for HRV recording [60].

HRV measurement was performed by recording the last 5 min of training and after finishing it for a further 10 min in a relaxed sitting position [32]. Due to the sudden change in HRV caused by the transition between training and recovery, the first 5 min of recovery were discarded [43], finally obtaining the values of 5 min at post-exercise for later interpretation.

The RR time intervals obtained from the HRV recording were later downloaded and analyzed using the Kubios HRV software (Version 3.3, University of Eastern Finland, Kuopio, Finland) [61]. Each record was previously analyzed to detect the possible presence of noise and abnormal beats, applying the corresponding filters if necessary.

During HRV measurement, RMSSD was recorded during exercise and at post-exercise. These data were later used to calculate the RMSSD-Slope by using the following formula as indicated by Naranjo-Orellena et al. (2019) [32]:

RMSSD-SLope = (RMSSD-postexercise − RMSSD-exercise)/time exercise

For an intensity of 60–75% of the maximum effort (coinciding with the intervals of the present study, 60–80%), the internal load score proposal according to the RMSSD-Slope value was: poor (<0.4); acceptable (0.4–2.6); very good (>2.6) [32].

#### 2.5.3. Sample Size

The sample size was calculated considering recent studies on the effects of BJ in RT [16,17]. A calculation based on a normal distribution was performed, with a power of 0.80 and a 2-tailed α level set to 0.05; the minimum number of participants required was estimated as 11.

#### 2.5.4. Randomization

A third person from outside the research team randomized all participants’ supplements (50% of participants took BJ and 50% took placebo at each visit). The online program https://www.randomlists.com/team-generator (accessed on 2 February 2020) was used.

### 2.6. Statistical Analysis

To assess the normality of the variables, Shapiro–Wilk tests were performed, and the equality of variance was contrasted with the Levene test. The comparison of the mean outcomes between BJ or placebo consumption was realized with a paired-samples *t*-test. For a practical significance of the results, effect size (ES) was calculated using Hedges *g* for repeated measures. ESs were considered to have large (ES > 0.8), moderate (ES = 0.8–0.5), small (ES = 0.5–0.2), or trivial (ES < 0.2) effects [62]. In addition, a general linear model for repeated measures was applied for the time–supplement interaction effect for the SBP, DBP, RMSSD first and final measurements. The Greenhouse–Geisser adjustment for sphericity was calculated. After a significant F-test, differences among the means were identified using pairwise comparisons with Bonferroni’s adjustment. A general linear model for repeated measures ES were calculated using partial eta squared (*η*^2^_p_), considering small to be under 0.25, medium 0.26–0.63, and large above 0.63 [63]. Significance was set at *p*-value < 0.05. SPSS software (Version 22.0, IBM SPSS Statistics for Windows, 2013; IBM Corp., Armonk, NY, USA) was used for the statistical analysis. The data are presented as mean ± SD.

## 3. Results

**Figure 1:**
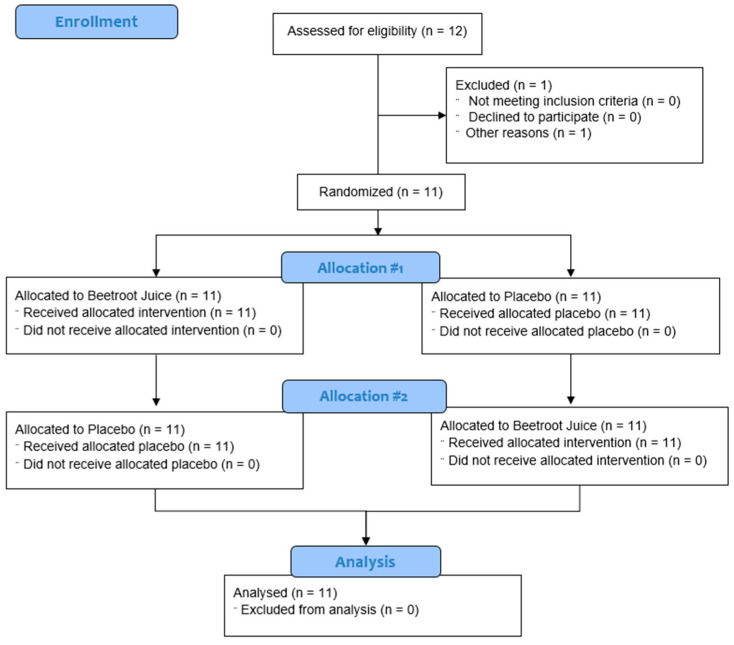
Flow diagram utilizing Consolidated Standards of Reporting Trials (CONSORT) guidelines.

Eleven men completed the two visits of the study protocol; one participant did not complete the study due to gastrointestinal problems. Figure 1 shows the flow diagram. Eleven participants were finally randomized, whose characteristics and anthropometrics data were published in a previous paper [52].

There were no differences in BP basal and post-exercise either in the HR mean or HR post-exercise. However, we did observe a difference in HR max, which was higher in the BJ group (Table 1). No differences were observed either in the interaction time x supplementation basal and post-exercise in the SBP (*p* = 0.444; *η*^2^_p_ = 0.054), or in DBP (*p* = 0.642; *η*^2^_p_ = 0.020).

**Figure 2:**
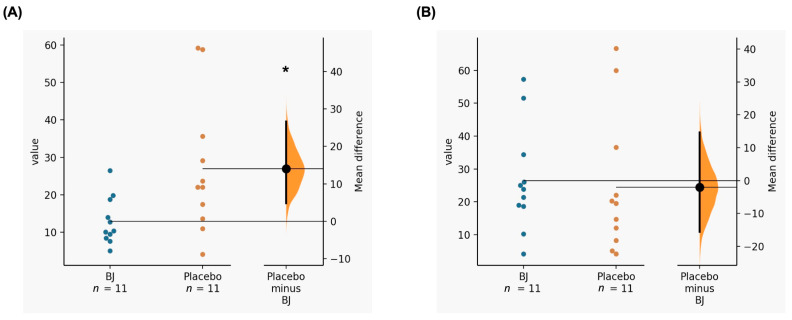
(**A**) Acute effects of BJ consumption compared to placebo on RMSSD during exercise; (**B**) Acute effects of BJ consumption compared to placebo on post-exercise RMSSD. BJ: beetroot juice. *: Significant differences (*p*-value < 0.05).

Differences were found in the RMSSD during exercise with a lower value in the consumption of BJ vs. placebo (BJ: 12.9 ± 6.3 vs. placebo: 26.9 ± 18; *p* = 0.023; ES = 0.999). There were no differences in the post-exercise RMSSD (BJ: 26.5 ± 16 vs. placebo: 27.7 ± 28.7; *p* = 0.914; ES = 0.050) (Figure 2). There was an interaction time x supplementation between the first and final measurement in RMSSD (*p* = 0.041; *η*^2^_p_ = 0.354). Therefore, there were differences in the RMSSD-Slope (BJ: 3 ± 3 (very good) vs. placebo: 0.5 ± 0.7 (acceptable); *p* = 0.025; ES = 1.104).

## 4. Discussion

This study aimed to investigate the possible effects of acute BJ supplementation on BP and variations in HR, HRV, and internal load during RT. The results of the study showed that BJ supplementation before training (120 min before) could produce changes in HRV, reducing RMSSD during exercise, but showing no changes in BP or RMSSD after exercise. Therefore, it seems that BJ could reduce the internal load measured through the RMSSD-Slope during RT, thus obtaining an improvement in RT performance, mainly in muscular endurance, as previously published [52].

Although there was no effect compared to placebo, BJ has the potential to decrease BP, vascular resistance and myocardial oxygen demand in both recovering and exercising subjects [64]. Vanhatalo et al. (2010) conclude that BJ reduces only SBP [65], whereas Webb et al. (2008) indicated that reductions in both systolic and diastolic BP were observed, respectively, 2.5 and 3 h post BJ supplementation [66]. Furthermore, systolic BP remained decreased after 24 for hours post ingestion, while diastolic BP returned toward baseline [67]. As a whole, these data invite us to think that BJ or nitrate-rich supplementation diet is more suitable to changes in systolic BP than diastolic. The result of this decline in BP can attenuate the O_2_ cost of an increment in work rate by 20% especially the younger one is. In fact, Stanaway et al. (2019) have described that SBP was reduced in young and older adults following BR supplementation, while DBP was reduced only in the older ones [68].

A possible explanation for the results of the present study may be the reactive oxygen and nitrate species (RONS) produced by RT [69]. Due to the antioxidant effect of NO_3_^−^ and betalains [70,71,72], it could be theorized that the lack of effect in our study is due to the oxidation of these components of the BJ carried out by the RONS produced during the RT. There is some controversy in the mechanism by which BJ reduce BP, because some reviews [9] and studies [65,68] suggest that this effect is due to NO_3_^−^ content, but a recent systematic review and metanalysis carried out by Bahadoran et al. (2017) [73] highlights that this effect could be NO_3_^−^ independent and may be related to other bioactive components such as betalains or other antioxidants [74,75].

RT has been probed to decrease acute parasympathetic modulation regardless of the age [76]. In the present study, it was shown how the BJ consumption modified the maximum HR and decreased RT-intensive exercise-mediated RMSSD in trained young men, reducing internal load compared with a control group. Similar to this, Benjamin et al. (2020) found that beetroot extract accelerates the return of parasympathetic modulation during recovery after an RT protocol in healthy adult men [77]. Although changes in HRV during aerobic exercise have been studied, an increase in HRV was observed during the day [78,79]. Carrijo et al. (2021) conclude that a single dose of BJ, independent of NO_3_^−^ content, does not change aerobic exercise-mediated responses in HRV indexes in time, frequency, and non-linear domains in hypertensive postmenopausal women [80], which is aspect not studied in trained men during RT.

In this regard, the BJ group had a significantly lower RMSSD during exercise than the placebo group. Exercise enhances the activity of the sympathetic nervous system [81,82] while reducing the vagal tone especially as the HR and exercise workload increase [83]. With RMSSD being a measurement of parasympathetic activation [84], besides the fact that the BJ group accumulated a greater total number of repetitions [52] and achieved a significantly higher HR than the placebo group, it could explain the reduced RMSSD during exercise in this group. Anyway, there were no differences between groups in post-exercise RMSSD, and the BJ group showed an increase in RMSSD-Slope between groups, which would mean a decrease in the internal training load in the BJ group [32,37]. A possible explanation for this could be the effect of NO on the autonomous nervous system, which could inhibit the sympathetic activity while increasing the vagal outflow [85,86] and thus enhance the recovery after exercise.

Despite the fact that, to our knowledge, no other clinical trials have studied the effects of BJ during RT in HRV. The relationship between HRV, sport and the utilization of some sport supplements has been analyzed before. In this regard, other commonly used supplements such us creatine limits the parasympathetic modulation of the RT exercise [87], whereas caffeine has contradictory results by increasing the parasympathetic modulation after anaerobic exercise [88] but delaying parasympathetic recovery after aerobic exercise [89]. Other not as common supplements such as the black thai ginger show a similar effect to caffeine in the response of autonomic nervous system to anaerobic exercise [90]. In this sense, studies that combine different supplements are required.

To our knowledge, this was the first study to evaluate the acute effects of BJ on HRV and internal load during resistance training. The results should be interpreted with caution. The main limitation of this study is the fact that only one measure of the HRV was realized in both groups and, with HRV being a very variable between days, it can bias the results of this trial. We suggest that future research on this topic should replicate HRV measurements not only to know the acute effects but also chronic ones. Another possible limitation to the study is the exercise order selection. Due to the back-squat exercise, which was performed previous to the bench press, and because of the back-squat involving a larger muscle volume [91,92], the accumulated neuromuscular fatigue and metabolic by-products [93] could cause different interpretation of the results if HRV was measured after back-squat instead of bench press.

## 5. Conclusions

In conclusion, no differences were found in BP between groups, but changes in HRV were found. Specifically, a decrease in RMSSD during exercise was observed with BJ consumption. It was accompanied with no differences in RMSSD post-exercise and an internal load reduction measured by RMSSD-Slope after BJ consumption while performing better on the muscular endurance test. Therefore, we conclude that BJ is a useful supplementation tool increasing the parasympathetic regulation in RT and thus decreasing the internal load.

## 6. Practical Applications

HRV measurement is a practical tool that can give a broader physiological understanding of sports performance. The effects of certain nutritional substances and sports supplements can affect the autonomic modulation of cardiovascular function. According to the results of the present study on the reduction in internal load derived from HRV parameters, it seems that BJ could be a suitable strategy to reduce exercise-derived fatigue even with a higher volume of training during RT.

## Figures and Tables

**Table 1:** Systolic and diastolic blood pressure basal and post-exercise, and heart rate during resistance exercise.

Variable	Beetroot Juice	Placebo	*p*-Value	ES
Systolic BP Basal (mmHg)	133 ± 22.2	129.5 ± 20.9	0.641	0.156
Systolic BP post-exercise (mmHg)	122 ± 11.1	124.7 ± 10.2	0.472	0.244
Diastolic BP basal (mmHg)	72.4 ± 11.7	76.7 ± 8.1	0.271	0.411
Diastolic BP post-exercise (mmHg)	68.8 ± 7.6	71.5 ± 11.6	0.285	0.265
HR mean (ppm)	129.3 ± 11.8	125.6 ± 12.5	0.208	0.293
HR post-exercise (ppm)	106.6 ± 12.5	101.8 ± 16.9	0.161	0.311
Maximum HR (ppm)	174.6 ± 9.1	168.5 ± 10.2	0.022 *	0.607

BP: blood pressure; ES: effect size; HR: heart rate. *: significant differences (*p*-value < 0.05).

## Supplementary Materials

The following supporting information can be downloaded at: https://www.mdpi.com/article/10.3390/nu14235119/s1, Supplementary Material S1: checklist CONSORT.
